# The efficacy of intravenous paracetamol versus dipyrone for postoperative analgesia after day-case lower abdominal surgery in children with spinal anesthesia: a prospective randomized double-blind placebo-controlled study

**DOI:** 10.1186/1471-2253-13-34

**Published:** 2013-10-22

**Authors:** Esra Caliskan, Mesut Sener, Aysu Kocum, Nesrin Bozdogan Ozyilkan, Semire Serin Ezer, Anis Aribogan

**Affiliations:** 1 Department of Anesthesiology and Reanimation, Faculty of Medicine, Baskent University, Ankara, Turkey; 2 Department of Pediatric Surgery, Faculty of Medicine, Baskent University, Ankara, Turkey; 3 Baskent University Faculty of Medicine, Adana Teaching and Medical Research Center, Baraj Yolu, 1. Durak, No: 37, 01110 Seyhan, Adana, Turkey

**Keywords:** Pediatric spinal anesthesia, Paracetamol, Dipyrone, Postoperative pain

## Abstract

**Background:**

A multimodal and preventative approach to providing postoperative analgesia is becoming increasingly popular for children and adults, with the aim of reducing reliance on opioids. We conducted a prospective, randomized double-blind study to compare the analgesic efficacy of intravenous paracetamol and dipyrone in the early postoperative period in school-age children undergoing lower abdominal surgery with spinal anesthesia.

**Methods:**

Sixty children scheduled for elective lower abdominal surgery under spinal anesthesia were randomized to receive either intravenous paracetamol 15 mg/kg, dipyrone 15 mg/kg or isotonic saline. The primary outcome measure was pain at rest, assessed by means of a visual analog scale 15 min, 30 min, 1 h, 2 h, 4 h and 6 h after surgery. If needed, pethidine 0.25 mg/kg was used as the rescue analgesic. Time to first administration of rescue analgesic, cumulative pethidine requirements, adverse effects and complications were also recorded.

**Results:**

There were no significant differences in age, sex, weight, height or duration of surgery between the groups. Pain scores were significantly lower in the paracetamol group at 1 h (*P* = 0.030) and dipyrone group at 2 h (*P* = 0.010) when compared with placebo. The proportion of patients requiring rescue analgesia was significantly lower in the paracetamol and dipyrone groups than the placebo group (vs. paracetamol *P* = 0.037; vs. dipyrone *P* = 0.020). Time to first analgesic requirement appeared shorter in the placebo group but this difference was not statistically significant, nor were there significant differences in pethidine requirements, adverse effects or complications.

**Conclusion:**

After lower abdominal surgery conducted under spinal anesthesia in children, intravenous paracetamol appears to have similar analgesic properties to intravenous dipyrone, suggesting that it can be used as an alternative in the early postoperative period.

**Trial registration:**

Clinical Trials.gov. Identifier: NCT01858402.

## Background

Acute pain is one of the most unpleasant experiences of childhood, and is generally a consequence of injury, illness, or medical intervention [1]. Acute pain management in children is increasingly characterized by multimodal or preventative approaches. The former comprises a combination of drugs and techniques such as non-steroidal anti-inflammatory drugs (NSAIDs), opioids, paracetamol, and regional or neuraxial anesthesia.

During the last three decades, spinal anesthesia has become increasingly popular in pediatric practice, but its use is not universal and some patients, anesthesiologists and surgeons still prefer general anesthesia. Nonetheless, spinal anesthesia is an easy and effective technique that provides highly effective analgesia, and sympathetic and motor block in the lower part of the body [2].

Single-shot intrathecal blocks have a limited duration of action, depending on the local anesthetic agent used. Therefore, a combination of other analgesics is required to treat pain when the spinal block wears off. These include opioids, non-opioids, and adjuvant drugs. Opioids are often used to treat moderate to severe pain in children; however, their use is limited by undesirable side effects such as cardiovascular, central nervous system and respiratory depression, itching, urinary retention, and nausea and vomiting [3].

Many studies in adults and children have shown that non-opioid analgesics such as paracetamol, NSAIDs, and dipyrone exhibit an opioid sparing effect and improve the quality of postoperative analgesia [4]. Paracetamol is the most commonly used analgesic to treat mild and moderate postoperative pain in children [1]; an intravenous formulation is now available. Dipyrone is a pyrazoline-derived analgesic drug with antipyretic, anti-inflammatory, and spasmolytic properties. The reliability and efficiency of intravenous paracetamol has been demonstrated in several clinical studies [5-7], and some have compared the antipyretic effectiveness of paracetamol with dipyrone in young children with fever [6,7].

The aim of this study was to assess the analgesic effect of intravenous paracetamol and dipyrone for postoperative pain relief in children undergoing spinal anesthesia for lower abdominal surgery.

## Methods

The Institutional Ethics Committee of Baskent University Faculty of Medicine approved the study protocol [Project no: KA 09/06]. Written, informed consent was obtained from the parents or guardians of each patient. A total of 63 healthy, ASA physical status I and II children, aged 8 to 15 years, undergoing elective lower abdominal surgery were enrolled in this study. Exclusion criteria included any known contraindication for spinal anesthesia, such as increased intracranial pressure, hemorrhagic diathesis and infection at the puncture site. Those with a known history of allergy to the study drugs were excluded.

Intraoperative monitoring consisted of non-invasive blood pressure measurement, electrocardiogram, pulse oximetry, and end-tidal carbon dioxide measurement using a nasal adapter. After peripheral intravenous access had been obtained, an infusion of 0.45% NaCl in 5% glucose was administered at 5 ml/kg/h. All patients had been premedicated with intravenous midazolam 0.05 mg/kg before lumbar puncture and supplemental oxygen 3 l/min was administered. Children who were anxious or felt uncomfortable after premedication or during surgery received further sedation with intravenous boluses of propofol 0.5–1.0 mg/kg.

Lumbar puncture was performed with the children in the lateral decubitus position with the midline approach with a 26-gauge spinal needle (Atraucan; Braun, Melsungen, Germany) at the L4–5 intervertebral space. After free flow of CSF was observed, 0.3 mg/kg 0.5% hyperbaric bupivacaine was injected. The extent of sensory block was tested with a pinprick method, and the degree of motor blockade was assessed using the modified Bromage scale [6,8].

The children were randomized to one of three groups according to a pre-generated randomization scheme created by the web site Randomization.com.

(http://www.randomization.com).

**Figure 1:**
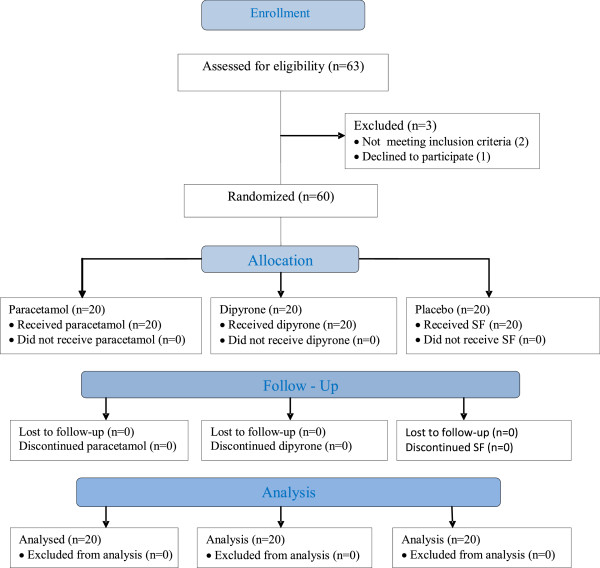
Flow chart of the study design.

After spinal anesthesia, children in Group *Paracetamol* received intravenous paracetamol (Perfalgan™ Bristol-Myers Squibb GmbH, München, Germany) 15 mg/kg premixed with 0.9% NaCl to a total of 50 ml. Group *Dipyrone* received intravenous dipyrone (Adepiron®, Adeka, Istanbul, Turkey) 15 mg/kg premixed with 0.9% NaCl to a total of 50 ml. The *placebo* group, received 50 ml 0.9% NaCl intravenously (Figure 1). All study drugs had been prepared by a researcher (MS) blinded to the treatment protocol, and were administered using identical infusion pumps over 15 min by another researcher (AK) blinded to the content of the infusion.

Intraoperative hemodynamic data, operation types, duration of surgery and degree of motor blockade were recorded. After surgery, the children were transferred to the post-anesthesia care unit (PACU), and the time taken for the block to recede by two segments was recorded. A consultant anesthetist (EC) blinded to the group to which each patient had been assigned performed all subsequent assessments.

The intensity of postoperative pain at rest was assessed using a visual analog scale (VAS) (where 0 represented no pain and 10 the worst pain ever experienced) at 15 and 30 minutes, and 1 h, 2 h, 4 h and 6 h after surgery. Pain at rest was the primary outcome measure.

Each patient’s sedation level was measured using a graded scale (0 = fully awake, 1 = awake but drowsy, 2 = sleeping, but arousable by light touch or speech, and 3 = sleeping, not arousable) at the same time. Postoperative pain was treated according to the same protocol in all groups. Intravenous pethidine 0.25 mg/kg was administered as rescue analgesia when VAS exceeded 4 out of 10, until the pain score was less than 4 or to a total dose of 1 mg/kg. Time to first administration of pethidine and cumulative pethidine consumption during the first six postoperative hours were recorded as secondary outcome measures.

Sedation levels, and all adverse effects including hypotension (>20% decrease in systolic blood pressure from baseline), bradycardia (heart rate <60 beats/min), respiratory depression, and nausea or vomiting were recorded and treated until the child was discharged. Children were discharged from the PACU when they were fully awake, hemodynamically stable, breathing satisfactorily and able to flex their hips. The time taken to be ready for PACU discharge was also recorded.

### Statistical analysis

*A priori* power analysis was performed based on the likely difference in the subjects’ postoperative pain scores evaluated by VAS. Sample size calculation was informed by the findings of a study previously undertaken at our institution [9], which indicated a likely 30% reduction in pain scores reported by subjects receiving an analgesic drug compared with placebo. We identified that the highest pain scores tended to be reported 2 h after surgery, equating to a maximum difference between means of 14 mm on the VAS with a standard deviation (SD) of 15 mm. Assuming a two-tailed type I error α = 0.05 and a power of 0.80, *a priori* analysis suggested 19 patients would be needed in each group (Power and Precision™ Biostat Inc., Englewood, NJ). All subsequent statistical analyses were performed using SPSS for Windows software (Statistical Package for the Social Sciences, version 17.0, SSPS Inc., Chicago, IL, USA). The analysis of variance test (ANOVA) was used for numerical and continuous variables to assess differences between groups. Homogeneity of variances was calculated using Levene’s test and Lilliefors significance correction. *Post hoc* analyses were performed with the Bonferroni test. Either the chi-square or Fisher’s exact test was used to analyze categorical variables when appropriate. Data are presented as mean ± SD, median with range or number of cases. A *P* value <0.05 was considered statistically significant.

## Results

Sixty-three patients were invited to participate. During screening, two patients were found not to meet the inclusion criteria, and one patient’s parents declined consent. A total of 60 patients constituted the study population (Figure 1). There were no statistically significant differences between the groups with regard to age, sex, weight, type of operation, duration of surgery, or anesthesia (Table 1).

**Table 1:** Characteristics of study groups

	**Paracetamol (n = 20)**	**Dipyrone (n = 20)**	**Placebo (n = 20)**
Sex (male/female) (n)	20 / 0	18 / 2	17 / 3
Age (years)	8 (7-15)	8 (7-13)	8 (7-15)
Weight (kg)	26 (20-60)	26,5 (20-50)	30 (20-51)
Height (cm)	136 (124-160)	130 (120-158)	132 (115-158)
Duration of surgery	40.7 ± 11.6	44.5 ± 13.7	42 ± 12.9
*Type of surgery* (n)			
Circumcision	8	8	7
Inguinal herniorrhaphy	4	4	4
Hydrocoelectomy	1	2	3
Orchiopexy	3	2	3
Hypospadias repair	1	2	2
Phlenoidal sinus	1	1	2
Varicocele	2	2	1

Data are expressed as medians (minimum-maximum) for age, weight and height, absolute numbers for gender and type of surgery, and mean ± standart deviation for duration of surgery.

**Figure 2:**
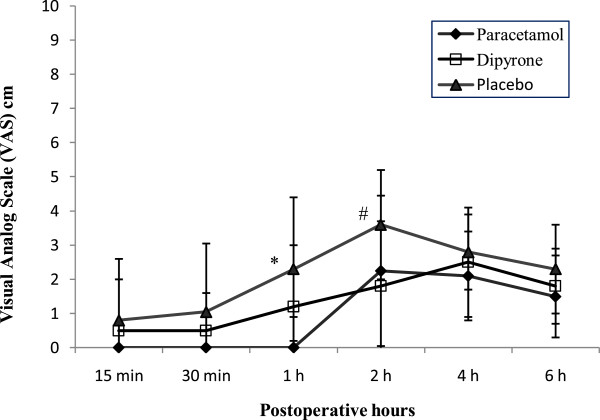
**Visual analog pain scores in the first 6 h after surgery.** Values shown are mean ± standard deviation. *P = 0.030 paracetamol *versus* placebo^, #^*P* = 0.010 dipyrone *versus* placebo.

Visual analogue scale (VAS) scores were statistically significant between Group *Paracetamol* and the *placebo* group after 1 hour (*p* = 0.030) and between Group *Dipyrone* and the *placebo* group after 2 h (*p* = 0.010)*.* There was no statistically significant difference in pain scores at any other times. The pain scores recorded on emergence from spinal anesthesia and in the first 6 hours after surgery are shown in Figure 2.

**Figure 3:**
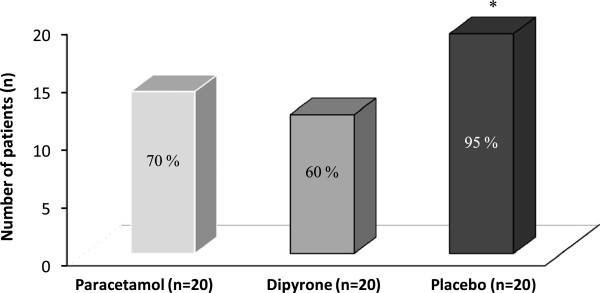
**Rescue analgesic requirements in the first 6 h after surgery.** Values are shown are number and proportion. h: postoperative hours. ^*^P = 0.037 paracetamol *versus* placebo, ^*^P = 0.020 dipyrone *versus* placebo.

The number of patients who received rescue analgesia was significantly lower for the *Paracetamol and Dipyrone* groups compared with the *placebo* group (paracetamol *versus* placebo, *p =* 0.037; dipyrone *versus* placebo, *p* = 0.020) (Table 2). Intravenous pethidine was given to 14 (70%) patients in the *Paracetamol* group, 12 (60%) in the *Dipyrone* group and 19 (95%) in the *placebo* group (Figure 3).

**Table 2:** Intraoperative parameters in each groups

	**Paracetamol (n = 20)**	**Dipyrone (n = 20)**	**Placebo (n = 20)**
Number of patients requiring additional sedation	7	8	9
Local anesthetic volume (ml)	1.7 ± 0.6	1.6 ± 0.4	1.8 ± 0.5
Time to two segments regression of block (min)	75 ± 15	74 ± 12	73 ± 14
Number of patients requiring rescue analgesia n (%)	14^*^(70%)	12^#^(60%)	19 (95%)
Time to first dose of rescue analgesic (hour)	2.9 ± 1.3	2.1 ± 1.3	1.8 ± 1.3
Total dose of rescue analgesic (mg)	9.5 ± 5.9	10 ± 3.4	10.6 ± 4.7
Time to ready for discharge from PACU (min)	75 ± 18	74 ± 21	72 ± 24

Data are expressed as mean ± standart deviation and absolute number of cases (n). *P =0.037 paracetamol *versus* placebo, #*P* = 0.020 dipyrone *versus* placebo.

The mean time to administration of rescue analgesia was 2.9 ± 1.3 h in the *Paracetamol* group, 2.1 ± 1.5 h in the *Dipyrone* group, and 1.8 ± 1.3 h in the *placebo* group, but these differences were not statistically significant (Table 2). There were also no significant differences in cumulative pethidine consumption or sedation scores between the three groups at any time point. Duration of stay in and time to meet the discharge criteria for the PACU, and time taken for regression of the sensory block and recovery of the motor block were broadly similar between the groups. There were no significant changes in hemodynamic parameters from baseline in either active treatment group. No episodes of hypotension or respiratory depression were recorded intraoperatively or in the PACU. Bradycardia was observed in two patients in each group (Table 3). There were also no significant differences in the incidence of postoperative nausea, vomiting, requirement for antiemetic rescue medication or any other perioperative adverse events between the groups (Table 3). No episodes of agranulocytosis were reported.

**Table 3:** Perioperative and postoperative adverse events

	**Paracetamol (n = 20)**	**Dipyrone (n = 20)**	**Placebo (n = 20)**
Hypotension	0	0	0
Bradycardia	3	3	3
Nausea	3	3	4
Vomiting	2	2	2
Respiratory depresion	0	0	0
Urinary retention	0	0	0

Data are presented as number of patients (n).

## Discussion

We evaluated the efficacy of intravenous paracetamol and dipyrone for administered as postoperative analgesia after day-case lower abdominal surgery in children. We found that postoperative pain scores were significantly lower in the active treatment groups in the first and second postoperative hours when compared with placebo, but there were no statistical differences in pain scores between the three groups at other times. We also found that the proportion of patients requiring rescue analgesia was significantly lower in the active treatment groups compared with placebo; however, there were no statistical differences in cumulative analgesic consumption.

The historic view that young children neither respond to, nor remember, painful experiences to the same degree as adults is no longer thought to be true [1]. Most premature neonates are capable of experiencing pain, and under treatment of pain results in harmful physiological and behavioral responses that have long-lasting negative consequences on the developing nociceptive systems [8,10]. Mather and Mackie [11] reported that 40% of children undergoing surgery experienced moderate to severe postoperative pain, and that 75% received inadequate pain treatment [12,13].

It has been reported that providing inadequate analgesia to infants, preverbal children, and adolescents results not only in short term physiologic disturbance but also longer term behavioral changes, particularly during immunization [10,14,15]. It is now recognized that optimal postoperative pain management is essential for children, and that this should begin before surgery by providing children and their parents with information about the planned surgical procedure [16].

Treatments for postoperative pain include drugs such as opioids, NSAIDs, paracetamol and dipyrone, as well as regional anesthetic techniques such as spinal and caudal anesthesia. The provision of adequate pain control must be balanced against the risk of the side effects of analgesics.

Neuraxial anesthesia in children is safe, provided appropriate care and attention are paid. Spinal anesthesia modifies the neuroendocrine stress response, ensures a more rapid recovery, and may shorten hospital stay with fewer opioid-induced side effects [17]. Furthermore, spinal anesthesia provides profound analgesia with minimal physiologic perturbations or side effects [17], and there is a growing interest in using the technique for surgery in preschool and school aged children [18]. Most studies in these populations have focused on specific procedures; none has examined the efficacy of paracetamol or dipyrone for pain relief after spinal anesthesia has worn off.

The main limitation of spinal anesthesia is the variable and relatively short duration of the block obtained with a single-shot local anesthetic injection technique [2]. Adding parenteral or enteral analgesic drugs such as opioid or NSAIDs is one means of overcoming these problems and thereby increasing the quality and duration of analgesia. Opioids are often used to provide effective postoperative analgesia; however, undesirable side effects may frequently be observed as a consequence of their use. Ideally a non-opioid analgesic such as paracetamol, an NSAID or dipyrone should be used to provide effective pain relief to minimize the need for opioids [19]. The safety of analgesic therapy has improved considerably as this multimodal opioid-sparing approach has been adopted; however, there are limited data concerning the efficacy of these regimes in the early postoperative period after surgery in children [20].

Paracetamol is commonly used as an analgesic and antipyretic in pediatric practice. It primarily acts centrally, where it is a potent antipyretic and mild analgesic. In recent years, an intravenous formulation has been introduced, and its safety and pharmacokinetic profile have been established for children as young as 1 month of age [12,21]. Dipyrone, a pyrazoline derivate, has antispasmodic, antipyretic and anti-inflammatory effects. It is widely used as an injectable non-opioid analgesic in several European and South American countries, where it has gained popularity due to the low incidence of adverse reactions (although like some other opioids it carries a low risk of agranulocytosis) [22]. Grundmann and colleagues stated that very large prospective studies would be needed to determine the true incidence of agranulocytosis [23]. A study comparing the adverse effects of paracetamol, diclofenac, aspirin and dipyrone reported that dipyrone was relatively well tolerated and shared many of the potential side effects of NSAIDs [22]. In our study, no incidences of agranulocytosis were reported.

Paracetamol may be preferred to dipyrone in children owing to its equal analgesic potency and lower risk of adverse effects. Several clinical studies have confirmed that the analgesic efficacy of intravenous paracetamol is comparable with that of NSAIDs or dipyrone in orthopedic, abdominal, and breast surgery in adults [22,24,25]. Landwehr and colleagues [24] reported that dipyrone and paracetamol had similar analgesic potencies after retinal surgery, whereas Grundmann and colleagues [23] demonstrated that the analgesic potency of dipyrone was superior to paracetamol during the first two postoperative hours after lumbar disc surgery. To date, the only study comparing the use of paracetamol with dipyrone in children examined their antipyretic properties [6]. Ours is the first study to examine their analgesic properties, and found that they have broadly similar efficacy in the first six hours after lower abdominal surgery in children.

Various studies have compared the analgesic efficacy of intravenous paracetamol with other analgesic agents such as tramadol, NSAIDs, and pethidine in children [5,19]. Alhashemi and colleagues reported that intravenous paracetamol was as efficient an analgesic as intramuscular pethidine in children undergoing tonsillectomy [19]. Paracetamol provided significantly greater analgesic effect than a placebo after orthopedic surgery in children [26], and a relatively large intravenous dose improved pain control after major spinal surgery in children and adolescents [27].

Our results demonstrate that paracetamol and dipyrone provide effective and comparable analgesia 1 h and 2 h after surgery, respectively, based on a lower and equivalent VAS scores compared with placebo. Although more patients in the placebo group required rescue analgesia, pain scores and cumulative pethidine consumption in the three groups were not significantly different in the first six postoperative hours. This concurs with the findings of Cakan and colleagues, who found that paracetamol 1 g administered intravenously at the end of surgery and 6 hourly thereafter did not reduce opioid requirements but improved the quality of pain relief [28].

A possible explanation for these findings might be that the sample size in our study was not large enough to detect subtle differences. Our power analysis was based on the assumption that detecting a 30% decrease in pain score on a VAS was clinically relevant and should be our primary outcome measure, but this might not be sufficiently large to detect pethidine consumption. This might also explain the lack of a significant difference in time to first analgesic requirement. It is notable that in previous studies of the use of bupivacaine for spinal anesthesia in children, the time to first analgesic requirement in the placebo group was 168 ± 70 min, which was broadly similar to our findings [29]. It is likely that in some patients the spinal anesthesia was still effective in the early postoperative period. It has been suggested that if the duration of preemptive analgesia extends into the postoperative period, it is more likely that pain hypersensitivity can be avoided [3]. Also, the provision of pethidine as rescue analgesia is likely to have influenced the subsequent low pain scores in all groups.

In our opinion, both paracetamol and dipyrone can be considered effective and equipotent analgesics in the early postoperative period, but that the small differences when compared with placebo indicate that they both only exert a mild effect.

The use of opioid analgesics is an important risk factor for postoperative nausea and vomiting (PONV). A reduction in the amount of systemic opioids would be expected to reduce the incidence of PONV and other common side effects. In our study, the use of pethidine was significantly lower in the paracetamol and dipyrone groups compared with the placebo group, but there was no statistically significant difference in the incidence of PONV between the groups. This may be a consequence of broadly similar cumulative pethidine doses administered to participants in each group.

## Conclusion

In conclusion, our study showed that preemptive administration of intravenous paracetamol or dipyrone provided effective pain control and reduced pethidine requirements in the first and second postoperative hours compared with placebo. Paracetamol appears to be at least as effective as dipyrone for pain relief, and it can be recommended as a viable alternative to dipyrone after lower abdominal surgery in children.

## Abbreviations

NSAIDs: Non-steroidal anti-inflammatory drugs; VAS: Visual analog scale; PONV: Postoperative nausea and vomiting.

## Competing interests

The authors have no conflicts of interest to declare.

## Authors’ contributions

All those listed as authors contributed to the preparation of the manuscript. Each listed author participated in the work to the extent that they can all publicly defend its content. EC coordinated the study and wrote the draft manuscript. MS participated in study design and undertook the statistical analysis. AK, NB, SSE and AA conceived the study, participated in its design, and coordinated the drafting of the manuscript. All authors have read and approved the final version.

## Pre-publication history

The pre-publication history for this paper can be accessed here:

http://www.biomedcentral.com/1471-2253/13/34/prepub
